# Experiences of lifestyle change among women with gestational diabetes mellitus (GDM): A behavioural diagnosis using the COM-B model in a low-income setting

**DOI:** 10.1371/journal.pone.0225431

**Published:** 2019-11-25

**Authors:** Lorrein Shamiso Muhwava, Katherine Murphy, Christina Zarowsky, Naomi Levitt

**Affiliations:** 1 Department of Medicine, Faculty of Health Sciences, University of Cape Town, Cape Town, South Africa; 2 Chronic Diseases Initiative for Africa, Cape Town, South Africa; 3 University of Montreal, Hospital Research Centre and University of Montreal School of Public Health, Montreal, Canada; 4 School of Public Health, Faculty of Community and Health Sciences, University of the Western Cape, Cape Town, South Africa; Brown University, UNITED STATES

## Abstract

**Background:**

Lifestyle change can reduce the risk of developing type 2 diabetes among women with prior gestational diabetes mellitus (GDM). While understanding women’s lived experiences and views around GDM is critical to the development of behaviour change interventions to reduce this risk, few studies have addressed this issue in low- and middle- income countries. The aim of the study was to explore women’s lived experiences of GDM and the feasibility of sustained lifestyle modification after GDM in a low-income setting.

**Methods:**

This was a descriptive qualitative study on the lived experiences of women with prior GDM, who received antenatal care at a public sector tertiary hospital in Cape Town, South Africa. Nine focus groups and five in-depth interviews were conducted with a total of thirty-five women. Data were analysed using content analysis and the COM-B (Capabilities, Opportunities, Motivations and Behaviour) model to identify factors influencing lifestyle change during and beyond the GDM pregnancy.

**Results:**

The results suggest that the COM-B model’s concepts of capability (knowledge and skills for behaviour change), opportunity (resources for dietary change and physical activity) and motivation (perception of future diabetes risk) are relevant to lifestyle change among GDM women in South Africa. The results will contribute to the design of a postpartum health system intervention for women with recent GDM.

**Conclusion:**

Our findings highlight the need for health services to improve counselling and education for women with GDM in South Africa. Support from family and health professionals is essential for women to achieve lifestyle change. The experience of GDM imposed a significant psychological burden on women, which affected motivation for lifestyle change. To achieve long-term lifestyle change, behaviour interventions for women with prior GDM need to address their capability, opportunity and motivation for lifestyle change during and beyond pregnancy.

## Background

Low- and middle-income countries (LMICs) have the highest mortality due to non-communicable diseases [1]. In 2016, diabetes was the second leading cause of mortality in South Africa, accounting for 5.5% deaths [2]. In addition, the country is among the top five in the African region with the highest prevalence of diabetes (9.3%) [3]. The prevalence of gestational diabetes mellitus (GDM) in South Africa, currently estimated to be 9.1% [4] is increasing along with the rise in obesity among women of reproductive age [4–6]. For the majority of women, GDM resolves after the affected pregnancy [7] but there is a 7-fold increased risk of developing type 2 diabetes in the 10 years thereafter [8]. According to a recent systematic review, the risk for progression to type 2 diabetes is highest within 3 to 6 years of the GDM pregnancy [9]. There is consistent evidence demonstrating that in at-risk populations lifestyle change mainly diet and physical activity, can reduce the risk of developing type 2 diabetes [10–13] and among GDM women, continuation of lifestyle changes after a GDM pregnancy can prevent progression to type 2 diabetes [7, 14]. In addition, studies have shown that postpartum follow-up, continued monitoring and ongoing support for lifestyle change are critical in motivating women to sustain efforts to change their behaviour. GDM therefore provides a unique window of opportunity to educate women on their future risk of type 2 diabetes and to engage them on lifestyle change to prevent or delay progression to type 2 diabetes [15].

Several qualitative studies have explored women’s perspectives on GDM and lifestyle related behaviour change in North America and Europe [16–21]. However, studies on women’s lived experiences of GDM in LMICs are still lacking [22, 23]. Lifestyle change among low-income populations should be viewed in the broader socioeconomic context of high rates of poverty and unemployment, where food choices are limited by affordability [24] and physical inactivity is increasingly prevalent [3]. Although women with GDM achieve lifestyle change during pregnancy, sustaining these lifestyle changes is particularly challenging in the postpartum period [7, 25]. Common barriers to continued lifestyle change postpartum include poor understanding of health information, financial constraints, low perception of risk of developing type 2 diabetes, lack of motivation, emotional stress, personal and family adjustment to the baby [25–28]. Such primary research is recommended by the Medical Research Council framework as an important first step in the development of evidence-based interventions [29]. An in-depth understanding of contextual factors is also critical if such interventions are to be not only well informed, but realistic and feasible [29, 30].

The present study is a component of the formative research phase for the development and evaluation of an integrated health system intervention for women with GDM in the South African public health system. The main study, (IINDIAGO) [31] is a complex intervention trial aimed at reducing the subsequent risk of progression to type 2 diabetes among women with GDM. The planned intervention starts in pregnancy and continues post-partum. It leverages the scheduled 6-week immunisation visit for the baby to conduct an Oral Glucose Tolerance Test (OGTT) for the post-GDM mother and to provide ongoing social and professional support to facilitate long-term lifestyle changes. The aim of this study was to explore women’s lived experiences of GDM and their views around the feasibility of sustained lifestyle modification after GDM, with a view to inform the development of a tailored behaviour change intervention. The specific objectives were to gain insight into women’s knowledge, understanding and interpretation of GDM, as well as their perceptions and lived experiences regarding lifestyle modification and health behaviour change (past achievements and difficulties; perceived barriers; factors that influence their health-related behaviour during pregnancy and in the postpartum period).

## Methods

### Study design

The study was a descriptive qualitative study to understand the lived experiences of women who had GDM, how they interpreted or made meaning of the experience and their perspectives on the potential for lifestyle change in their context. Focus group discussions were used to explore women’s knowledge and experiences of GDM and reveal their understanding and evaluation of their capacity for behaviour change. The group setting encouraged conversation among participants revealing different views whilst also enabling collection of data on shared perspectives. Challenges with recruitment necessitated in-depth interviews instead of focus groups with five participants, which therefore still allowed us to explore their lived experiences.

### Theoretical framework

The chosen theoretical framework for the development of the IINDIAGO intervention was the COM-B model of behaviour change outlined in the Behaviour Change Wheel [32]. The COM-B (Capabilities, Opportunities, Motivations and Behaviour) model incorporates context in understanding behaviour and developing behaviour change interventions, while providing a systematic method for analysing the target behaviour and then characterizing interventions based on the behavioural diagnosis [32]. Behaviour is a result of the reciprocal interaction between the three fundamental components; Capability, Opportunity and Motivation [32]. The COM-B model can be used to structure an analysis into the barriers to and enablers for behaviour change in a given context, thereby ensuring that intervention developers set realistic behaviour change targets [33, 34]. This model has been used in the context of GDM to develop effective health communication content for the STAR MAMA program for low-income post-GDM Latina women in the US [35]. In the present study, the COM-B model was used to guide the analysis of the focus group data and provide a theoretical framework for understanding GDM women’s’ capacity for lifestyle change and the available opportunities and barriers in their environments.

### Setting

In South Africa, government funded public sector health services cater to more than 80% of the population, who cannot afford the high costs of medical insurance and private health services [31]. The study was conducted at a public sector, tertiary referral hospital, which provides health services to patients residing in Cape Town and other surrounding areas of the Western Cape province. Once diagnosed with GDM, women are referred from their primary health care facility or a district hospital to the tertiary hospital, where they attend a dedicated antenatal diabetes clinic until delivery. The majority of women are referred from Midwife Obstetric Units in Cape Town’s low-income, peri-urban townships. The women seen at this hospital are predominantly coloured (mixed ancestry) and black African who speak mainly Afrikaans and isiXhosa, as well as English. Antenatal care for women with GDM is provided by a team of health care providers including obstetricians, endocrinologists and nurses.

### Participant selection

A folder audit was conducted to identify women with GDM who had delivered at the tertiary hospital in Cape Town between 2014 and 2015. Information regarding the GDM diagnosis and general medical history was used to purposively select potential participants that met the inclusion criteria. The following were eligible: women who had been diagnosed with GDM between 2014 and 2015; had received antenatal care at the tertiary hospital; delivered a live baby and who did not need treatment for diabetes at discharge. Eligible participants were contacted telephonically and invited to participate in focus group discussions at a private seminar room on the hospital premises. Reasons for refusal were recorded in a communication log. Depending on their preference, women could select a suitable day to attend a focus group discussion from provided options. Focus groups were scheduled with between five to ten women per focus group. Reminders were sent by telephone and text message a day before and on the morning of the scheduled focus group to confirm attendance. To improve recruitment, we scheduled focus groups on weekends to accommodate women who were working during the week, organised focused groups at local community venues for those women who could not travel to the hospital and allowed women to bring their infant along to the focus group. When fewer than three women turned up for a scheduled focus group, the researchers took the opportunity to conduct individual in-depth interviews.

### Data collection

A focus group guide was used in the discussions and consisted of open-ended questions and probes. The main topics for discussion were knowledge and attitudes regarding GDM, experience of health care during GDM pregnancy, experience of health care in post-partum period, lifestyle modification during and after pregnancy and finally, attitudes to the proposed postpartum intervention for GDM women. Participants were encouraged to raise other issues of interest to them that related to the broad topic of the discussion. The focus groups and in-depth interviews allowed for discussions with women until saturation was reached. Two female researchers (LSM and KM) trained in qualitative research techniques conducted the focus groups in English. A third co-facilitator (BMD); a diabetes nurse who was conversant in the two local vernacular languages, isiXhosa and Afrikaans, was present to assist participants who preferred to express themselves in their native language. The responses were then interpreted into English for the benefit of the wider group. The co-facilitator was also responsible for taking notes, observing group dynamics and monitoring the audio recorder to increase dependability of the data collected. Each focus group lasted between one and two hours or until data saturation was reached. Verbal consent to participate in the focus group was obtained telephonically. At the start of each focus group and interview, the researchers introduced themselves and explained the purpose of the study. Written informed consent was then obtained from each participant and all participants were given the opportunity to ask questions. Participants were reimbursed for their transport costs and given a supermarket grocery voucher for their time as well as a locally-developed recipe book for preparing affordable healthy meals. Healthy snacks and refreshments were served during intervals to encourage informal social interaction among participants. Focus groups and interviews were audio recorded and transcribed verbatim. Transcripts were stored in a locked cabinet with restricted access.

### Data analysis

Data analysis followed the methods of qualitative content analysis as described by Elo & Kyngäs [36]. The analysis process was both inductive in that some categories were derived from the data to understand women’s lived experiences of GDM and how they interpreted it and deductive in that it was driven by the broad conceptual categories theorised to be the precursors of behaviour change in the COM-B model [32]. The pre-existing, conceptual categories of the COM-B model provided a lens through which to view and interpret the data.

Using this model served to answer the key research questions of a) What was the potential for change, given the barriers and opportunities perceived and experienced by this population of GDM women? and b) What kind of intervention was needed to meet their needs and enhance their potential for change?

The first author (LSM) is a public health researcher and is familiar with the SA maternal health context. The second author (KM) is a qualitative researcher whose research work focuses on the development and evaluation of behaviour change interventions for NCDs. The third author (CZ) is a medical doctor and medical anthropologist, with substantial clinical and health systems research experience in the SA setting. The last author (NL) is a clinical endocrinologist in the public health sector with strong interests in NCDs in the African context. All four authors are female. For triangulation and to facilitate a robust analysis, LSM and another researcher, worked independently in coding the transcripts. As a first step, all transcripts were read for overall familiarization with the data. The transcripts were then re-read and annotated to understand meaning from the participant’s perspective. The data was then systematically organised and abstracted through a process of open coding and generating conceptual categories. LSM and the second coder met frequently to discuss and compare their analyses, resolve discrepancies and came to an agreement on a common coding framework to apply across all the data. KM was present in the discussions between the data coders to ensure credibility of the findings. This coding framework was further developed and refined through continued collaboration between the researchers as the analysis proceeded. For the purposes of this manuscript, the data was analysed according to the categories of the COM-B model; *Capability*, *Opportunity* and *Motivation*. Other emergent themes were also identified in the data but are to be the focus of other papers.

### Ethical approval

Written informed consent was obtained from each participant in the focus groups and interviews. Ethics approval for the study was obtained from the University of Cape Town (HREC: 946/2014) and Université de Montréal (CR CHUM: 2018–7091, 17.128-ID). Permission to conduct the folder audit was obtained from the relevant hospital authorities.

## Results

Nine focus groups (N = 30) and five in-depth interviews were conducted between March and June 2016. Eight focus group discussions were held at the hospital study site and one at a community venue. Twenty-three women declined participation and fourteen accepted participation in the focus groups but could not attend due challenges with transportation, time and availability of child care. Participants’ demographic information is summarised in Table 1. Our sample consisted of black African and ‘coloured’ (mixed ancestry) women. The majority of participants were married (60%) and unemployed (77%).

**Table 1:** Summary of participants’ demographic characteristics (N = 35).

Variable	Frequency	%
**Age (years)**		
25–29	7	20
30–34	15	43
35+	13	37
**Marital Status**		
Single	13	37
Married	21	60
Divorced	1	3
**Employment Status**		
Employed	6	17
Unemployed	27	77
Student	2	6
**Home Language**		
English	11	31
Xhosa	10	29
Afrikaans	10	29
Shona	1	3
French	3	9

**Fig 1:**
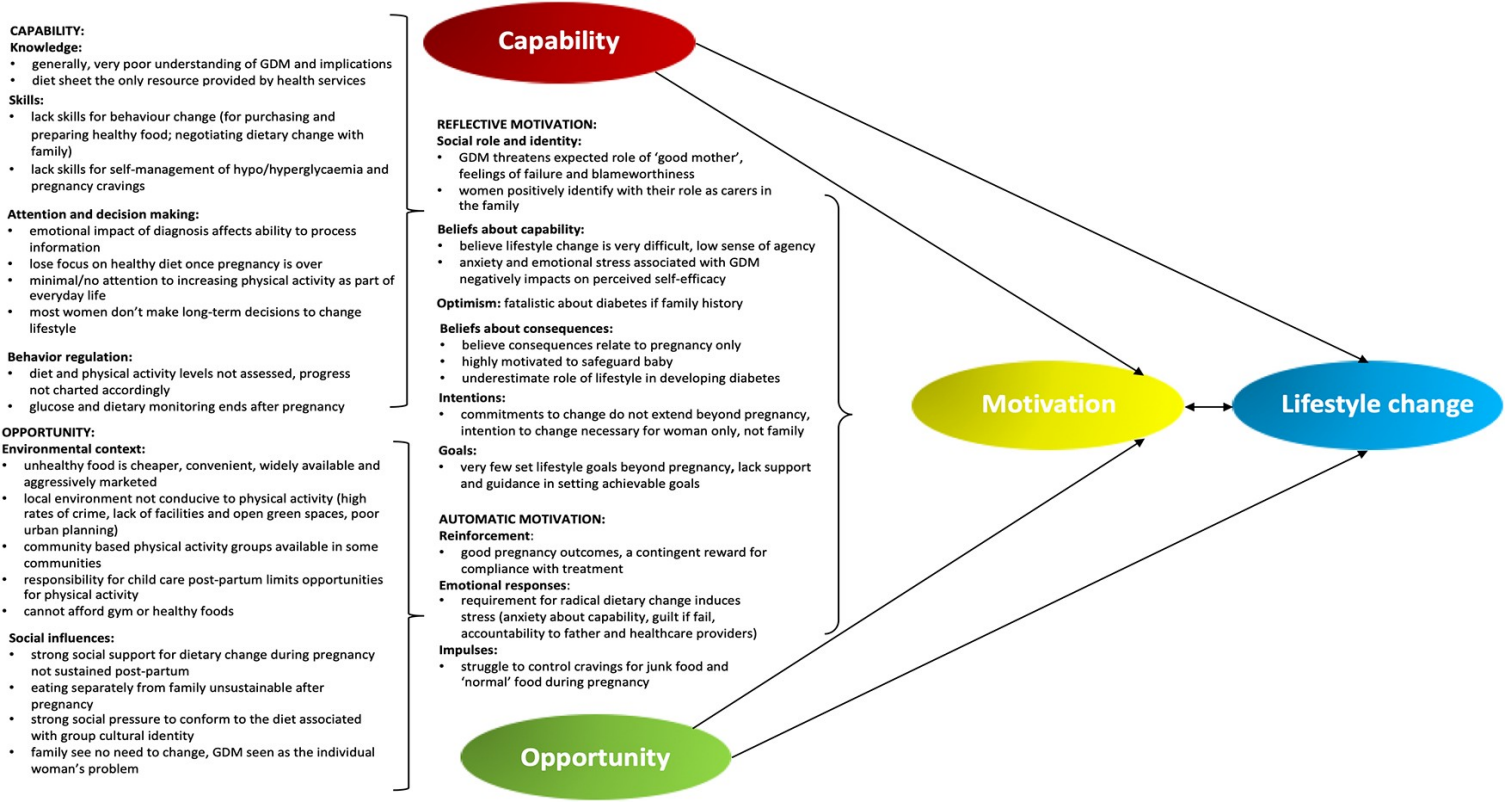
Findings from focus group discussions mapped according to the Theoretical Domains Framework (TDF) of the COM-B model framework for understanding behaviour [38].

The results are organised according to the constructs of the COM-B model namely; Capability, Opportunity and Motivation, which are necessary pre-conditions for desired behaviour to occur. Fig 1, adapted from Howlett et al [37], represents the focus group findings in relation to the Theoretical Domains Framework (TDF) of the COM-B model [38].

### Capability

Capability can be described as the individual’s ‘physical and psychological capacity to engage in the activity concerned’ [32]. Physical capability refers to the physical skills required to achieve a desired behaviour whereas psychological capability is the capacity to engage in the thought processes necessary for behaviour change [30]. Both physical and psychological capability can be improved through intervention or training [32].

#### Psychological capability

(i) Limited knowledge and understanding of GDM

Generally, the women demonstrated limited knowledge and understanding of GDM. They expressed a strong desire for the GDM clinic to provide more education and counselling as part of their routine clinical care. They felt that this was especially important during their first visit at the hospital following their referral, because this was a time of heightened anxiety and confusion. There was a general sense that due to high patient numbers, health care providers did not have the time to explain the GDM diagnosis to them. Although women spoke of the high standard of clinical care they received, they were dissatisfied with the lack of counselling and education and were left feeling frustrated by the lack of opportunity to ask questions to help them clear up their confusion.

*There’s no proper interaction between you and the person you’re sitting with*, *to tell you*, *‘this is the reason why you’ve got this*.*’ There’s no explanation*, *so you can imagine my confusion*.

*(Focus Group 4*, *Participant 14)*

*I didn’t understand why I can’t have normal food*, *because no one said to me*, *‘You have Diabetes’*

*(Focus Group 4*, *Participant 16)*

One woman explained how she had to strongly assert herself in order to get the answers she needed:

*I started snooping through my file and searching the internet to understand what the terms meant*, *which caused frustration and that pent-up feeling in me*. *You’re just left in the dark there until you throw a tantrum*. *You’ve got to raise the roof in order for them to actually give you a decent sit-down conversation*.

*(Focus Group 6*, *Participant 20)*

Whilst most women relied on health care providers as a primary source of information, a couple with access to resources such as the internet and dieticians in private health services took the initiative to seek out more information in order to gain a better understanding of their condition.

*I told myself*, *it’s the first-time pregnancy with Gestational Diabetes*, *and I’m going to try to get as much information as I can*. *I also did a lot of research on how it is caused and how I’m actually going to deliver the baby*

*(Focus Group 9*, *Participant 35)*

Other participants were more knowledgeable regarding GDM due to their experience of having a family member or partner with diabetes. These women felt less anxious on receiving the diagnosis as diabetes was familiar to them;

*I understand Diabetes because my mother has Diabetes*. *I understand what you have to eat and that sometimes there are days where you don’t feel well because your sugar levels are not okay*.

*(Focus Group 4*, *Participant15)*

Women described being so emotionally overwhelmed by the GDM diagnosis that they were unable to process and understand the limited information they did receive during the initial consultation. They suggested that during the diagnosis consultation, providers be sensitive to the emotions that women may experience and instead, leave the provision of information and advice on how to manage GDM for a subsequent consultation. As one participant recalled:

*There wasn’t room for me to ask questions*. *I was shown how to prick myself [insulin injections] but I was still in so much emotion; I couldn’t even recall how often she said I had to do it*.

*(Focus Group 1*, *Participant 4)*

The education materials provided by the hospital did not help fill the gap in knowledge. Typically, women received a one-page diet sheet. This information was limited in scope: prescribing what dietary changes needed to be made but offering no detailed guidance about *how* to change behaviour. When directly questioned about physical activity, the majority of women reported that physical activity was not mentioned or emphasized by health care providers as part of recommended lifestyle changes.

*They didn’t explain that [physical activity]*. *They just said lifestyle change*, *but they didn’t explain*, *like*, *exercises and things like that*, *no*

*(Interview 1*, *Participant 5)*

Only a few women recalled being advised to do some light to moderate physical activity by the dietician.

*They did say something about exercise*, *just to take a brisk walk*, *maybe thirty minutes*, *but not too much*, *but just so that you can exercise*

*(Focus Group 9*, *Participant 35)*

This lack of understanding of GDM sometimes resulted in denial in accepting the GDM diagnosis and resistance to treatment;

*They said they have to put me on Insulin*, *and I was very upset*, *I was very cross*. *I even told myself*, *I’m not going to use the Insulin*.

*(Focus Group 3*, *Participant 12)*

In contrast to this respondent’s evident anger about the lack of counselling and information, it was clear that some women made adjustments to their lifestyle (in particular by making dietary changes) regardless of their lack of understanding of GDM and its implications.

*My health would have been in bad condition*, *and it would also have affected my son*, *so whatever they used to tell me*, *I used to follow*. *If they tell me*, *do this at this time*, *you have to eat this*, *I used to follow*.

*(Focus Group 8*, *Participant 32)*

#### Physical capability

(ii) Physical discomfort during pregnancy

A few women reported that they had engaged in some leisurely physical activity such as walking in the neighbourhood or parks, yoga and attending a private or community gym, but that this became more difficult towards the end of the pregnancy, due to physical discomfort.

*In the last trimester*, *I was huge*, *and I looked uncomfortable*, *because I’m short*. *People thought I was carrying twins*, *because of my size and my belly*. *It was very uncomfortable*

*(Focus Group 1*, *Participant 4)*

Another woman said that she felt too anxious to do her usual walking once she was full term as she was afraid of the onset of labour:

*We had a nice path*, *so I could just walk down there*, *just a bit*, *because I didn’t want to stretch myself too far*, *and anything could happen*

*(Focus Group 1*, *Participant 4)*

### Opportunity

Opportunity refers to ‘all the factors that lie outside the individual that make the behaviour possible or prompt it’ [32]. These are factors in the environment that encourage or discourage achieving behaviour change, which can be physical (related to time, access to resources, affordability of resources, actual physical environmental barriers, existence of cues) or relating to the social context (including interpersonal influences that can cause individuals to change or not change their beliefs, attitudes, feelings, or social norms, culture, social pressure, expectations of others, group identity) [32].

#### Physical environment

(i) Opportunities for physical activity

In general, the majority of women in this study did not engage in leisure time physical activity outside of their daily activities such as chores and travel to work because of concern for personal safety, due to high levels of crime and violence.

*I stopped going to the gym is because I had to walk alone at night when I’m finished*, *we’re mostly just girls there I’m too scared to walk around there; the shooting and stuff*.

*(Focus Group 5*, *Participant 17)*

Some women mentioned that there were available opportunities in their communities, which they had utilised.

*We have a community exercise programme in a local hall*, *so*, *I started exercising*. *It’s aerobics*, *running*, *it’s everything*. *It was hardcore*. *I felt like I was the odd one out with all this weight*,

*(Focus Group 1*, *Participant 2)*

*There’s a centre*, *where they give gym to the ladies that stay at home*, *every Thursday morning*. *There is a qualified instructor who comes to Mitchells Plain*, *and the City Council pays her*.

*(Focus Group 2*, *Participant 6)*

Post-partum, it was difficult for these women to continue with physical activity as certain facilities did not accommodate children.

*We moved houses and I had to change the gym*. *At that gym you can’t take your children so that was difficult for me*. *My husband is at work*, *so where must I leave my children*, *and then I just stopped*.

*(Focus Group 4*, *Participant 16)*

Women also suggested that the health services could increase opportunities for physical activity:

*Activities need to be provided*, *like how about a walkabout in the hospital for the patients*. *I mean*, *for the guys who have just had a Caesar*, *they need to get mobilised as soon as possible*

*(Focus Group 6*, *Participant 19)*

(ii) Influence of prevailing food environment

Two participants who worked in catering, described how continual exposure to unhealthy food in their work environments made sticking to the healthy eating recommendations particularly difficult:

*The thing is that where I used to work*, *I was in charge of the kitchen*. *I didn’t know what to eat*. *I was eating junk*, *any food which I see*. *I didn’t know how to control myself*, *you know*

*(Focus Group 8*, *Participant 32)*

The one participant described how support from the head chef helped her to overcome this barrier:

*It was stressful because I had cravings and I was hungry all the time*. *My head chef said ‘No*, *I’m going to set up a diet for you*. *You’re not going to eat that food; you must think of your baby*.’

*(Focus Group 8*, *Participant 27)*

(iii) Affordability of healthy food

There was a general perception among respondents that healthy foods were more expensive than “ordinary foods”. While pregnant, the woman and her family were prepared to incur the extra expense of adhering to dietary recommendations to safeguard the pregnancy, but once the baby was born, it was understood that this extra cost could not be sustained. As most women were unemployed and financially dependent on their partners, they had limited decision-making power in relation to household expenditure.

*For me it’s expensive*, *because I don’t work*. *Before we had the baby*, *it was okay*, *because it was just the two of us*, *but now we’ve got more expenses for everything*. *It’s either I get his things or buy stuff for myself*. *I can’t do both of them*

*(Focus Group 4*, *Participant 14)*

Continuing with a healthy diet was also considered difficult post-partum because women believed that it required eating separately from the rest of the family. This was a more expensive way of eating, as well as being impractical when having to also cater for the rest of the family.

*When one goes shopping*, *you would normally buy what you need to make the normal pot of food [for the entire family]*. *But now you have to cater for yourself and the things aren’t cheap if you want to eat healthy and fresh*. *The sugary things are much cheaper than the healthier things*.

*(Interview 1*, *Participant 5)*

#### Social environment

(iv) Prevalent social norms

A high carbohydrate diet, the consumption of sugary beverages and fatty meat were perceived as part of a ‘normal’ diet and an aspect of cultural or community identity. As a result, dietary change during pregnancy was a major adjustment for some women, as it required shifting from the social norm.

*I was used to my cool drinks and things*, *it’s the type of home that you come out of*. *I was used to having cool drink or juice at supper and so it was a big lifestyle change that I had to do*.

*(Interview 1*, *Participant 5)*

*I would eat the food like any normal African eats*. *I would put a lot of sugar in my tea*. *Now I’m eating more healthy food*, *when I make chicken*, *I remove the skin*. *I boil the chicken and my milk is low fat*. *When I want to put sugar*, *I put very little brown sugar*.

*(Focus Group 2*, *Participant 8)*

(v) Importance of social support

All women emphasized the importance of having a supportive social environment to make lifestyle changes and indicated that support from partners, family, peers and health professionals was essential. Participants described how receiving encouragement and motivation from their partners and family in particular, aided them in making the necessary changes. Some family members changed their diet to support the expectant mother and others supported her by ensuring that she adhered to a healthy diet.

*My mom was my best supporter*. *If I bought chicken with my veggies*, *she will eat with me*.

*(Focus Group 8*, *Participant 30)*

*My family was very supportive*, *and my husband was very strict*. *He’ll watch me at restaurants and functions*. *They will always remind me*.

*(Focus Group 3*, *Participant 11)*

Women also recalled sharing experiences and tips with other pregnant women during their antenatal clinic visits or when hospitalized and how these relationships provided a sense of mutual support. Some women also expressed sincere appreciation for the compassion and support they received from healthcare providers during their pregnancy. Such social support and feelings of connection positively impacted on their perceptions of the quality of care they received and were clearly important in enabling them to adhere to treatment. For example:

*The professor gave me inspiring encouragement; and I think that is what kept me going*.

*(Focus Group 2*, *Participant 6)*

*They have a Sister; we called her the ‘Breast Sister’*. *They would call her up just to help [with breastfeeding] and she’s the loveliest person ever*. *She encouraged you and she would motivate you*.

*(Focus Group 4*, *Participant 15)*

This was not however the experience of all women: some recounted very negative experiences with healthcare providers:

*The sisters were very rude*. *We even had to remind them about getting our medication and food*. *They don’t have patience for us*. *I was always arguing with them*, *because they’re not on time to give you medication*

*(Focus Group 9*, *Participant 34)*

*The [nursing] staff are so stressed out there; they really don’t have the patience to deal with the patients*.

*(Focus Group 6*, *Participant 19)*

### Motivation

Motivation refers to ‘all the brain processes that energize and direct behaviour, not just goals and conscious decision-making’ [32]. This consists of reflective processes (i.e., conscious intent, goal setting and planning) and automatic processes (i.e. habits, reflex behaviours, impulses determined by external factors) involved in evaluating the potential consequences and benefits of carrying out or achieving the desired behaviour [32].

#### Reflective motivation

(i) Concern for the health of the baby

During pregnancy, concern for the health of the baby was reported as a strong motivating factor for adherence to prescribed medication. Although some participants reported anxiety and side effects associated with metformin and insulin, these were outweighed by the perceived benefits for their health and that of the unborn baby. Thus, they continued to take the medication for the sake of the baby;

*I remember the first time I took Metformin*, *I started shivering*. *It was like a cold shiver that I’d never experienced before*. *It was so bad*, *and I wanted to stop taking it*, *but I couldn’t because of the baby*, *you know*, *I had to consider the baby*.

*(Focus Group 4*, *Participant 15)*

*So right through the pregnancy I was on Insulin and Metformin*, *and it’s not an easy thing for us*. *It’s really not easy to inject yourself every time*.

*(Focus Group 8*, *Participant 29)*

For the majority of the women, adherence to treatment and the recommended lifestyle change during pregnancy were non-negotiable and a necessary sacrifice, even if they did not fully understand their condition.

*You don’t know how this [GDM] is affecting your pregnancy and what it’s going to do to the baby*, *you don’t know*, *you’re just going to do what you need to do for the safety of yourself and your baby*.

*(Focus Group 4*, *Participant 14)*

(ii) Fear of failure as a ‘good’ mother

One respondent expressed resentment towards some health care providers because of their judgemental and blaming attitudes:

*They make you feel like your body is failing*. *They say*, *‘listen*, *you ate wrong and it’s your fault if something happens to your baby.’*

*(Focus Group 1*, *Participant 2)*

Women discussed the challenges of caring for a new baby and how these impacted their sense of identity as mothers. One respondent described how struggling to breastfeed added to her feelings of failure as a ‘good’ mother:

*He couldn’t latch on and I felt so guilty*. *I waited till my husband went to bed every night and then I’d cry when he was sleeping*.

*(Focus Group 4*, *Participant 16)*

(iii) Prioritising own health after the pregnancy

Women described their intentions to focus on their own health after delivery and expressed a desire to continue the healthy lifestyle habits they had developed during pregnancy in order to reduce their risk of type 2 diabetes;

*During pregnancy your focus is your baby because you want everything to be okay with baby*. *So*, *after baby is born*, *you take more time also into your own life again*, *and then you realise*, *okay*, *I have to focus on me now*.

*(Interview 1*, *Participant 5)*

(iv) Desire to lose weight

Some women were unhappy with the fact that they had not returned to their pre-pregnancy weight and expressed a strong desire to continue with physical activity and a healthy diet for weight loss. Being overweight or obese was described as uncomfortable and an emotional burden;

*With weight also comes a lot of stress*. *I’m getting older and changing*. *I don’t do family functions a lot anymore*, *because where am I going to get an outfit*? *I’m always tired*, *because I’m overweight and I’m not enjoying myself the way I used to*

*(Focus Group 1*, *Participant 2)*

(v) Considering the next pregnancy

The desire for another baby motivated some women to continue with the lifestyle changes after the GDM pregnancy. They were also more prepared and equipped with the knowledge gained during pregnancy to sustain the behaviour changes postpartum.

*I’m really more health wise*, *because it’s very important*. *I do want another baby*. *That’s why I’m looking after myself*. *I go to gym every Thursday*.

*(Focus Group 2*, *Participant 6)*

#### Automatic motivation

(vi) Psychological vulnerability

Women’s feelings of motivation to make lifestyle changes were affected by their emotional responses to the GDM diagnosis. Having GDM led to feelings of fear, worry, anxiety and stress during pregnancy.

*It was very traumatising*, *and I cried for several days*. *It was my first baby*, *and I waited so long for this baby*. *I was scared*

*(Focus Group 8*, *Participant 30)*

Some women continued to experience psychological distress even after delivery, which affected their ability to cope with motherhood and continue lifestyle changes. When asked about postpartum follow-up care and diabetes screening, one woman reported being unable to attend the clinic for postpartum follow-up due to postpartum depression (PPD):

*I was in post-natal depression*. *I just didn’t feel like me*. *My husband couldn’t understand why I was so moody and edgy*. *I just couldn’t cope*.

*(Focus Group 5*, *Participant 15)*

Table 2 provides a summary of facilitating factors identified for developing capability, opportunity and motivation for lifestyle change during and after a GDM pregnancy.

**Table 2:** Summary of facilitating factors for developing capability, opportunity and motivation for lifestyle change during and after a GDM pregnancy.

*Capability*	*Opportunity*	*Motivation*
**Psychological capability** *Knowledge and understanding of GDM* Provision of counselling and education on GDM by health care providers Detailed educational materials teaching skills for lifestyle change Ability to engage with health care providers and ask questions Access to additional resources for further information Experience of caring for a family member or partner with diabetes **Physical capability** Tolerating physical discomfort and fatigue during pregnancy	**Physical opportunity** Access to affordable healthy food options Access to safe outdoor spaces for physical activity within their community Availability of personal time for physical activity after delivery **Social opportunity** *Professional support* Expert advice, encouragement, compassion and empathy from health care providers during pregnancy *Family support* Emotional and practical support from family and friends in making lifestyle changes Support from family in caring for the baby *Autonomy* Having negotiating power regarding family diet *Cultural influences* Healthy food incorporated into individual and group cultural identity Able to resist social pressure to eat unhealthy food at family gatherings and other social events Supportive social norms regarding physical activity	**Reflective motivation** *Concern for the health of unborn baby* Fear of stillbirth, deformities Adherence to treatment despite side effects for the sake of the baby Fear of delivery by Caesarean section Fear of failure as a mother *Concern for own health* Fear of developing type 2 diabetes post-partum Prioritising and valuing own health after pregnancy Desire for weight loss after pregnancy Intention to have another baby and fear of another GDM pregnancy **Automatic motivation** Ability to exercise self-control and resist unhealthy food during pregnancy Receiving support to address emotional responses to GDM diagnosis and for mental health issues (e.g.; anxiety & stress during pregnancy; postnatal depression)

## Discussion

To our knowledge, this is the first qualitative study in sub-Saharan Africa to explore the lived experiences of women with GDM as well as the feasibility of sustained lifestyle changes beyond pregnancy. Several key factors that influence women’s ability to implement lifestyle change were identified (Fig 1) and are discussed in relation to the COM-B model constructs and in the context of existing literature. These findings will contribute to the development of an appropriate and feasible behaviour change intervention for women with prior GDM to reduce the risk of developing type 2 diabetes among this population.

### Capability

A common finding was that lack of information from their health care providers impacted negatively on women’s capability to respond adequately to the GDM diagnosis and affected their adherence to treatment. Women with GDM in China [22] and Vietnam [39] also reported that the health information they received from health care providers was unclear, lacking detail and that they desired more information about GDM. Women’s poor knowledge of GDM is associated with poor understanding and therefore poor interpretation of health information [25]. For example, some respondents interpreted the fact that GDM resolves after pregnancy to mean that once the pregnancy was over, they could revert to their ‘normal’ unhealthy lifestyles. Poverty, poor maternal education and low health literacy all contribute to women’s poor comprehension of health information on GDM management [40]. In order to improve women’s psychological capability to respond to GDM, health services must prioritise the provision of sufficiently detailed health information at diagnosis, a view supported by systematic review findings on postpartum health care seeking behaviour among post-GDM women [26].

All women in this study were interested in understanding their GDM diagnosis and desired an opportunity to engage with health care providers to improve their knowledge and develop skills for self-management (e.g.; glucose monitoring and insulin administration) and behaviour change (e.g.; purchasing and preparing health meals). Given the very limited time available for counselling in our setting due to high patient numbers and resource limitations, GDM women should be provided with additional, comprehensive and culturally appropriate educational resources, that take varying literacy levels into consideration [26, 41]. It is noteworthy that the provision of such information alone would be insufficient to effect long term behaviour change [30] and is likely to be most helpful to women when delivered in the context of a supportive, personal interaction, which allows them to engage personally with the information and ask questions [7].

### Opportunity

Our findings that women with GDM have to overcome several barriers to lifestyle change in their physical and social environment are in agreement with studies with GDM women from high-income countries such as the United States and Ireland [12, 42, 43] and some LMICs [44]. During the GDM pregnancy, the woman and her family direct all financial resources towards her to ensure positive obstetric outcomes. However, financial responsibilities increase once the baby is born and opportunities for lifestyle change (i.e. finances, personal time) become more limited in the post-partum period with the added responsibilities of child care [7].

Another important finding was that our respondents were generally unaware of the physical and mental health benefits of physical activity. This has previously been reported by pregnant women from similar communities of low socioeconomic status in Cape Town [45]. This could be readily overcome by consistent counselling or messaging, but the barriers of a lack of attractive, local open outdoor spaces and high levels of crime in in their communities require different strategies. These include creating safer spaces within communities, introduction of physical activity programs by the health services, partner, family and community involvement and the use of media to raise awareness [46, 47]. The GDM pregnancy is also an opportune time for women to establish physical activity routines that can realistically be sustained beyond the pregnancy, for example; incorporating simple physical activity into their daily routine and in the postpartum period, easing back into physical activity with safe and light exercises that can be done while holding the baby.

The majority of women experienced strong social support from their families during pregnancy for dietary change. However, some women reported having some difficulty in persuading their partners and families to change the overall family diet. In SA, as in other patriarchal societies, women have limited negotiating power to significantly change the family diet. Further, the consumption of certain high caloric foods has become incorporated into group cultural identity and dietary change despite its benefits, requires deviation from social norms. The same social influence of family and friends on women’s diet has also been noted among non-pregnant women with type 2 diabetes in Soweto, South Africa [48]. Health behaviour change interventions should therefore target GDM women’s partners family members and social networks [34].

Expert advice and psychological support from health care providers during pregnancy were highlighted as critical. However, in the postpartum period, the absence of follow up left women feeling abandoned. It was interesting to note that women in this study found emotional release from sharing their experiences and valued the peer support derived from participating in the focus groups. The perceived withdrawal of support, which has also been found in studies conducted in the UK [18, 19] and Australia [49], resulted in some women reverting back to unhealthy lifestyle behaviours. Thus, ongoing support from health services during pregnancy and thereafter, that may be complemented by peer support groups [50, 51] should be considered.

### Motivation

Women’s initial emotional reactions to the GDM diagnosis of fear, worry, anxiety and stress [19, 51] illustrated their deep sense of responsibility for their baby. According to the literature, concern for the baby is the strongest motivating factor for behaviour change during pregnancy [18, 40] and in our study provided motivation to make major lifestyle adjustments at any cost, including resisting cravings for unhealthy food and enduring the side effects of medication. Some respondents described negative experiences with antidiabetic therapy in keeping with previous reports that women with GDM find insulin use overwhelming and burdensome [16, 41, 51]. Yet in comparison to dietary modification, others prefer insulin use to manage GDM [40].

The psychological impact of the GDM diagnosis may be currently underestimated by health services. As with some respondents in this study, GDM women in previous studies have reported feeling unsupported by their health care providers to face the overwhelming distress of the GDM pregnancy [22]. With a GDM pregnancy, women are often perceived as baby machines [19] closely scrutinised by their partners and health care providers, which adds to maternal anxiety. The diagnosis and experience of GDM in itself has been linked to postpartum depression (PPD) [52], which affects women’s motivation to sustain lifestyle changes. Although a GDM pregnancy can be considered a ‘prime teachable moment’ [34], this needs to be tempered with ‘caring GDM care’, which Ge et al., have described as humanistic care [22]. Findings from previous research suggest incorporating a clinically valid psychological assessment into the care for women with GDM to assess the impact of GDM on quality of life [53, 54]. In addition, increasing health care providers awareness of the emotional impact of GDM supported by targeted psychological interventions and provision of adequate information could help alleviate the anxiety and psychological distress associated with GDM and thereby enhance women’s psychological motivation for behaviour change [19].

In summary, women’s capability to make lifestyle changes was significantly impacted by lack of knowledge and understanding of GDM. Health care providers should be trained in patient-centred counselling methods (e.g., motivational interviewing) and provide women with adequate health information and appropriate educational resources to facilitate their developing practical strategies to achieve lifestyle changes. The limited resources in LMICs make it particularly pertinent to tailor physical activity and dietary recommendations to women’s social and environmental context. It is clear that social support plays a critical role in facilitating lifestyle change as lack of support in the postpartum period resulted in disruption of healthy behaviours made during pregnancy.

### Strengths and limitations

Our study has several strengths. The descriptive qualitative approach allowed for detailed insights into women’s subjective experience of GDM and their perceived capacity for behaviour change, while the COM-B model provided a validated theoretical framework on which to map the facilitators for developing capability, opportunity and motivation for lifestyle change, as a first step towards the development of an appropriate behaviour change intervention for post-GDM women in this setting. The COM-B model may also be utilised by health care providers and those involved in health policy planning [30, 33, 34].

We acknowledge some limitations of this study. Generalisability of the findings is limited due to the qualitative study design. Our sample consisted of women who received antenatal care at a single tertiary hospital in the Western Cape, were reachable and agreed to participate. However, the study site is one of two large tertiary hospitals that receive specialist referrals for GDM from primary care clinics and district hospitals across the Western Cape province. Both hospitals adhere to provincial and national guidelines for the management of GDM. The intent of the study was not generalizability of findings but rather to explore women’s perceived capacity for behaviour change within their context. Our study sample is representative, in terms of socioeconomic status, ethnicity and age, of women in this low-income urban setting. Lastly, the participants were at least one year postpartum, which may have introduced recall bias. On the other hand, it gave women sufficient time to reflect on their experiences of the GDM pregnancy. Despite these limitations, our findings are consistent with evidence in the literature and may be applicable in other low-income urban areas.

## Conclusions

Using a combined descriptive qualitative and theoretical framework (i.e.; COM-B) approach, this study elicited important insights into the lived experiences of women with GDM and the feasibility of lifestyle change in a low-income setting. The findings highlight barriers to capability, opportunity and motivation for lifestyle change. Consistent counselling and provision of appropriate educational resources are necessary to overcome these barriers. In order to achieve long-term lifestyle change, continued support from the health services, partners, family members and peers is essential.

## Supporting information

S1 Discussion Guide (DOCX)
