# Supplementary material for: Experiences of lifestyle change among women with gestational diabetes mellitus (GDM): A behavioural diagnosis using the COM-B model in a low-income setting
Source: PLoS One. 2019 Nov 25;14(11):e0225431. doi: 10.1371/journal.pone.0225431 (PMC6876752; doi:10.1371/journal.pone.0225431)
Supplement: S1 Discussion Guide — (DOCX) [file pone.0225431.s001.docx]

**Study title: Formative research for the development of an appropriate, acceptable and feasible intervention aimed at reducing type 2 diabetes risk in disadvantaged women after gestational diabetes in South Africa**

**Focus Groups (FG) with GDM women postpartum**

**Knowledge and attitudes regarding GDM:**

1. Can you explain what you understand by gestational diabetes?

Prompt: What is GDM? How is it diagnosed? How is GDM treated?

Prompt: In your understanding, what are the consequences of untreated GDM to your health and that of your unborn baby?

1. What do you understand about being at greater risk of developing type 2 diabetes after GDM?
2. What health problems are associated with type 2 diabetes?

**Experience of health care during GDM pregnancy:**

1. During your pregnancy did you receive any information or advice from the health care provider on lifestyle changes; diet; physical activity; alcohol etc.? If so, please explain who advised you and what they said.

Prompt: Did you receive any information to take home (pamphlets/booklets)?

Prompt: Did you seek any other information about managing GDM? For example from books or internet sites?

1. What concerns did you have during pregnancy about your health and your baby’s health?
2. What support did you receive - personal/emotional/practical from your family or friends during pregnancy?
3. How did you feel about the medical care you received from the health care workers (doctor/nurse) during pregnancy?

Prompt: The manner in which you were treated, the way they explained information to you

Prompt: In your view, how well controlled was your diabetes during pregnancy?

1. After delivery, what counselling did you receive from the health care worker on the importance of follow-up care and screening for diabetes?
2. Were you referred to another health facility after discharge from the hospital (eg; local clinic/CHC?) Please explain.

Prompt: Were you provided with a referral letter when you were discharged from the hospital?

**Experience of health care in post-partum period:**

1. Did you go for a test for diabetes 6 weeks after you delivered your baby? If yes, Where did you go for the test?

**If NO**: Did you go for a test for diabetes any time after that?

**If NO**: What were your reasons for not going for follow-up?

1. Where did your baby receive their immunizations?

Prompt: Private or public health facility? Which one? Who took the baby for the immunizations? (If not the mother: Why were you unable to go with your baby for immunizations?)

1. How did you feel about the care your baby received from the health care workers at the Well Baby Clinic?
2. Did you breastfeed your baby? If YES: For how long did you breastfeed your baby exclusively?

**If NO**: What were your reasons for not breastfeeding your baby exclusively/not breastfeeding?

**Motivation for lifestyle modification**

*During pregnancy:*

1. When you were diagnosed with GDM during pregnancy, what changes to your lifestyle did you make, if any?

Prompt: Changes around diet, physical activity, alcohol intake, smoking etc

Prompt: What motivated you to make those lifestyle changes?

1. What were the difficulties you experienced (barriers) to making these lifestyle changes?
2. What could have made it easier for you to make these changes (facilitators)?

*After pregnancy:*

1. How has being at greater risk of developing type 2 diabetes influenced your diet and physical activity behaviours after pregnancy?

Prompt: Have you continued with the lifestyle changes in your diet and exercise since the baby was born? What has motivated you to continue with these lifestyle changes after pregnancy?

Prompt: Which of the lifestyle changes have you tried but failed to continue with? What difficulties have you faced with trying to continue with the lifestyle changes after pregnancy?

Prompt: What would help/make it easier?

1. What personal/emotional/practical support did you receive from your family/friends to continue with the lifestyle changes after pregnancy? Please describe.
2. What are your thoughts and feelings about you current lifestyle?

**Attitudes to proposed post-partum intervention for GDM women**

*First explain the proposed intervention: We want to develop health services and an education programme for women with previous gestational diabetes that will help to prevent them from getting diabetes later. Our programme would offer tests for T2D, education and counselling at Well Baby clinics.*

1. What information or support would you have liked to have received to help you after your GDM pregnancy?

Prompt: If it had been available would you have come for a test for T2D 6 weeks after delivery?

Prompt: What do you think of having the programme at the Well Baby Clinic? How far is the nearest Well Baby Clinic from your home? (either distance in km/time travelled walking/car/public transport)

Prompt: What information/advice would you have liked to get after pregnancy? Would you have liked individual counselling/group counselling/health education materials or all of the above? Would you have preferred counselling by the nurse or community health worker? In the clinic or a community venue?

1. **Final question:** Is there anything you know now about GDM after your experiences, that could help someone newly diagnosed with GDM? If yes, please explain.
